# Physics-informed optimization of saturation-transfer MRI protocols using non-differentiable Bloch models

**DOI:** 10.1088/1361-6560/ae4285

**Published:** 2026-02-16

**Authors:** Beomgu Kang, Munendra Singh, Hyunseok Seo, HyunWook Park, Hye-Young Heo

**Affiliations:** 1 Divison of MR Research, Department of Radiology, Johns Hopkins University, Baltimore, MD, United States of America; 2 Department of Artificial Intelligence, Korea University, Seoul, Republic of Korea; 3 School of Electrical Engineering, Korea Advanced Institute of Science and Technology (KAIST), Daejeon, Republic of Korea; 4 F.M. Kirby Research Center for Functional Brain Imaging, Kennedy Krieger Institute, Baltimore, MD, United States of America

**Keywords:** Bloch equation, MR fingerprinting, non-differentiable model, optimization, saturation transfer

## Abstract

Saturation transfer MR fingerprinting (ST-MRF) is a quantitative molecular MRI method that simultaneously estimates parameters of free water, solute, and semisolid macromolecule protons. The accuracy of these quantification is highly dependent on the choice of acquisition parameters, and thus, the optimization of the data acquisition schedule is crucial to improve acquisition efficiency and quantification accuracy. Herein, we developed a learning-based optimization framework for ST-MRF, incorporating a deep Bloch equation simulator as a surrogate model for the forward Bloch equation solver to enable rapid simulations. Notably, the deep Bloch equation simulator overcomes the non-differentiability of the original model by enabling gradient computation during backpropagation within the physics-informed optimization framework, thereby allowing iterative updates of the acquisition schedule to minimize quantification error. In addition, the proposed method estimated an accurate ΔB_0_ map with the inclusion of a minimal number of scans to address B_0_ inhomogeneity. B_1_ inhomogeneity was corrected by providing a relative *B*_1_ map as an input to the quantification network. We validated our approach using Bloch–McConnell equation-based digital phantoms and further evaluated the performance of the proposed optimized ST-MRF framework in *in vivo* experiments. Our results showed that the optimal ST-MRF schedule outperformed other data acquisition schedules with regard to quantification accuracy. In addition, we enhanced the *in vivo* quantitative maps by correcting motion artifacts and suppressing noise using self-supervised learning techniques. The optimal ST-MRF approach could generate accurate and reliable multi-tissue parameter maps within a clinically acceptable time.

## 1. Introduction

Saturation transfer (ST) MRI enables the specific detection of semisolid and mobile macromolecules as well as tissue metabolites through a molecular or proton exchange process, including magnetization transfer contrast (MTC) from semisolid macromolecules (e.g. myelin) or bound water protons with broad lineshapes (very short *T*_2_, 10–100 *µ*s) and chemical exchange ST (CEST) from mobile proteins and tissue metabolites (relatively long *T*_2_, 10–100 ms) (Henkelman *et al* 1993, Ward *et al* 2000, van Zijl *et al* 2003). Amide proton transfer (APT) imaging, a variant of CEST-based molecular MRI, has demonstrated significant promise in imaging brain tumors, stroke, and other diseases (Leigh *et al* 2017, Heo *et al* 2017b, Zhou *et al* 2019, 2022, Heo *et al* 2023, Jiang *et al* 2023, Wang *et al* 2023). However, current CEST or APT imaging protocols and previously published works rely on acquiring a water saturation spectrum (the so-called Z-spectrum), which is inherently non-quantitative (Zaiss and Bachert 2013, Togao *et al* 2014, Jiang *et al* 2019, Meissner *et al* 2019, Lee *et al* 2020, Yin *et al* 2025). Consequently, the resulting APT images are termed APT-weighted images due to multiple contributions from upfield nuclear Overhauser enhancement, MTC, and water relaxation effects. Moreover, these weighted images are influenced by experimental parameters, such as RF saturation parameters, pulse sequence design, and CEST calculation metrics (Heo *et al* 2017a, van Zijl *et al* 2018, Heo *et al* 2019c, Sun 2021, Zhang *et al* 2023). Therefore, there is a growing interest within the CEST research community in quantifying proton exchange rates and concentrations to accurately evaluate molecular properties.

Recently, an MR fingerprinting (MRF) approach has been integrated into ST imaging to enable quantification of MTC and/or CEST parameters (Ma *et al* 2013, Cohen *et al* 2018, Heo *et al* 2019a, Perlman *et al* 2023, Weigand‐Whittier *et al* 2023, Singh *et al* 2025a). Various RF saturation and acquisition settings, including saturation field strength (*B*_1_), saturation pulse duration (*T*_s_), frequency offset (Ω), and relaxation delay time (*T*_d_), have been applied in a pseudo-random (PR) manner to generate unique tissue-specific signal profiles, or ‘fingerprints’. Subsequently, multiple tissue parameters (such as free water, MTC, and CEST) are decoded from these acquired fingerprints by solving an inverse Bloch McConnell equation, e.g. model-based fitting, dictionary or pattern matching, and deep-neural-network-based approaches (Heo *et al* 2019a, Kim *et al* 2020, Hamilton *et al* 2021, Kang *et al* 2021, Metzner *et al* 2021, Perlman *et al* 2022a, Singh *et al* 2023). Importantly, static *B*_0_ and transmit *B*_1_ field inhomogeneities can disrupt MRF signal profiles, resulting in inaccurate quantification of tissue parameters (Buonincontri and Sawiak 2016, Chen *et al* 2016, Ma *et al* 2017). RF saturation-encoded ST imaging protocols are especially susceptible to these field inhomogeneities, making the quantification of proton exchange rate and concentration challenging. Therefore, correcting for B_0_ and B_1_ variations is crucial to ensure the accuracy and robustness of ST-MRF.

**Figure 1.**
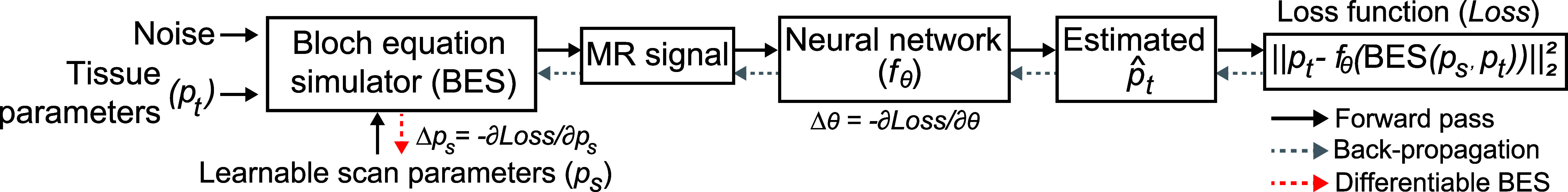
Physics-informed acquisition schedule optimization integrates Bloch equation simulations to synthesize MR signals, which are subsequently used for tissue parameter estimation and the computation of estimation loss. The scan parameters are updated via gradient descent method using the loss gradient with respect to the scan parameters, which is only feasible when the Bloch equation simulator is differentiable.

Importantly, within the MRF framework, optimizing the MRF acquisition schedule is critical to enhance the data acquisition efficiency and improve the accuracy of the tissue parameter quantification. Recent studies have introduced physics-informed deep learning optimization techniques aimed at minimizing quantification errors and accelerating imaging times by reducing the number of required data acquisitions (Perlman *et al* 2020, Kang *et al* 2022b, Cohen and Otazo 2023, Heesterbeek *et al* 2023). Learning-based optimizations optimize the acquisition sequence to directly minimize quantification errors of tissue parameters. *In vivo* studies have demonstrated that learning-based optimization of acquisition schedule (LOAS) outperformed indirect optimization methods (Kang *et al* 2022b), such as maximizing signal discrimination between tissue types and maximizing signal-to-noise-ratio (SNR) efficiency using the constrained Cramer–Rao lower bound (Cohen and Rosen 2017, Zhao *et al* 2019). Despite its superior performance, the current optimization framework remains limited to differentiable Bloch equation models, as the gradient of the quantification loss with respect to the scan parameter is required to update the scan parameters towards minimizing the loss (figure 1). As a result, the applicability of such gradient-based frameworks is restricted for a broad class of non-differentiable physical models. For example, this limitation arises in multi-pool pulsed ST acquisitions, where no closed-form solutions of the Bloch–McConnell equations exist and signal evolution must be computed numerically (Vladimirov *et al* 2025). Similar issues are also encountered in multi-pool models in which closed-form signal expressions are available, but the incorporation of extrapolated super-Lorentzian RF absorption rates for the semisolid macromolecular pool prevents the derivation of explicit gradients with respect to model parameters.

**Figure 2.**
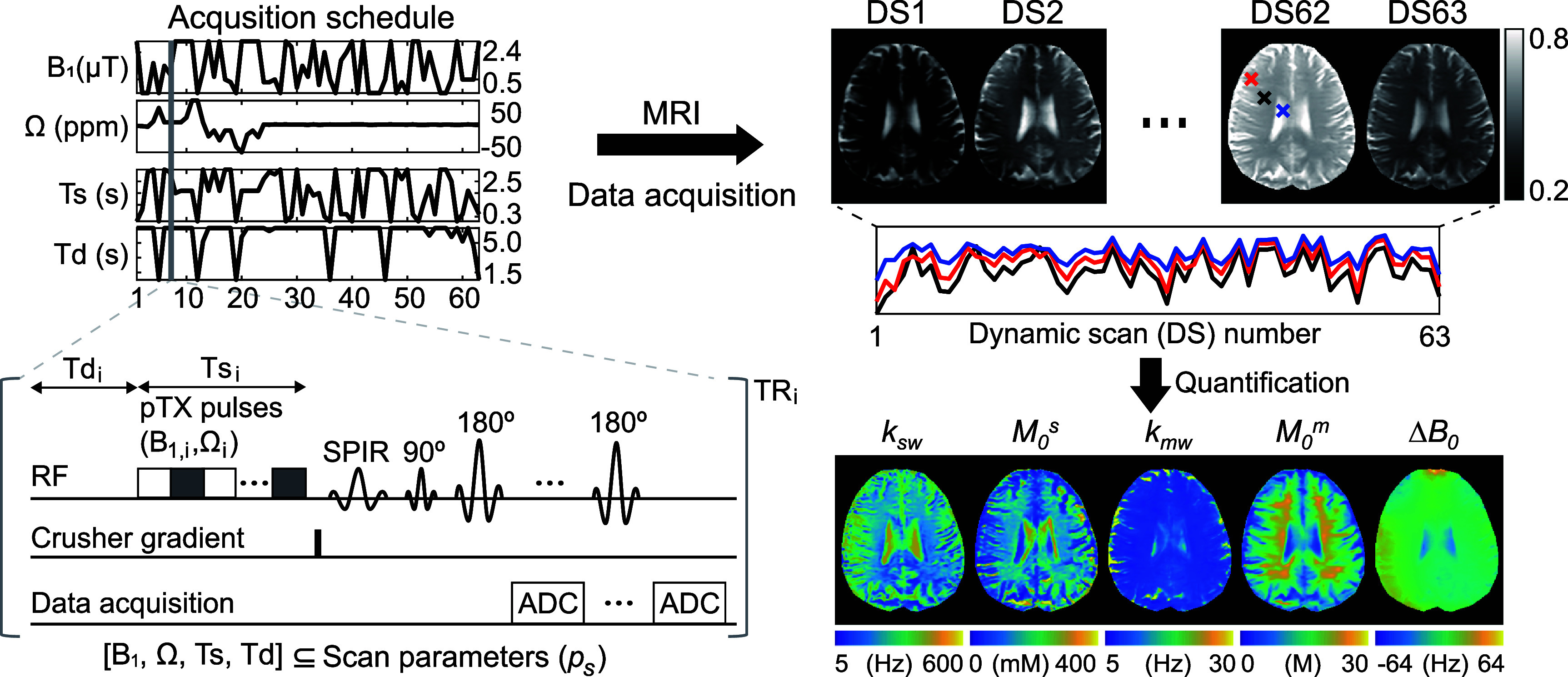
An illustration of saturation transfer MR fingerprinting (ST-MRF) acquisition. The acquisition schedule consists of four varying scan parameters, which are used to generate the ST-MRF signals. The acquired ST-MRF images are then used to estimate the quantitative tissue maps. For the MR sequence, continuous RF saturation was achieved using two-channel parallel transmission (pTX), with a crusher gradient applied at the end of the RF saturation to eliminate the residual transverse magnetization. Abbreviation: ADC, analog to digital converter; SPIR, spectral presaturation with inversion recovery; TR, repetition time.

Herein, we proposed a fast, efficient physics-informed acquisition schedule optimization framework for non-differentiable signal model by integrating a deep Bloch equation simulator, which enabled fast simulations and provided gradients for backpropagation. Previous learning-based MRF schedule optimization studies (Kang *et al* 2022b, Perlman *et al* 2022b) focused on two-pool exchange models that relied on relatively simple, differentiable analytical solutions to the Bloch equations. In contrast, we propose an optimization strategy using a three-pool exchange model that incorporates bulk water, solute, and semisolid macromolecule pools, along with an extrapolated super-Lorentzian RF absorption rate. This model enables improved characterization of biological tissue; however, it is non-differentiable. In addition, magnetic field-specific parameters (*B*_0_ and *B*_1_) were integrated in the optimization framework to improve the quantification of multiple tissue parameters. Finally, 3D multiple tissue parameter maps were obtained with a five-minute scan by optimal ST-MRF acquisition (figure 2). Meanwhile, ST-MRF images were enhanced by correcting motion artifacts and suppressing noise using self-supervised approaches that do not require ground truth clean data. The predictive deep-learning model and optimized MRF sequence were validated by means of Bloch–McConnell simulations and *in vivo* human brain scans at 3 T.

## 2. Theory

### 2.1. A transient-state three-pool exchange model

A three-pool exchange model (w: free bulk water proton pool, s: solute proton pool, and m: semisolid macromolecular proton pool) can be used to simulate ST effects, including MTC and CEST, in the presence of proton exchange, relaxation, and RF irradiation. It is assumed that the proton exchange between pool m and pool s is negligible compared to the exchange with pool w. The magnetization vectors of the three pools (***M***^w^, ***M***^s^, and ***M***^m^) can be described using the Bloch–McConnell equations as follows (Zhou *et al* 2004, Woessner *et al* 2005, Sun 2010, Heo *et al* 2016, 2024), as detailed in the appendix:

\begin{equation*}{\boldsymbol{M}} = \left({\boldsymbol{M}_0}\left(1 - {{\mathrm{e}}^{ - R_1^{\mathrm{w}}{T_{\mathrm{d}}}}}\right) + {\boldsymbol{A}^{ - 1}}{\boldsymbol{B}}\right){{\mathrm{e}}^{\boldsymbol{A}{T_{\mathrm{s}}}}} - {\boldsymbol{A}^{ - 1}}{\boldsymbol{B}}.\end{equation*}

The longitudinal magnetizations for each pool are simulated based on the given tissue parameters and RF scan parameters, which include saturation strength (*B*_1_), frequency offset (Ω), saturation time (*T*_s_), and relaxation delay time (*T*_d_). It is worth noting that differentiability is lost when the RF absorption rate is modeled using a super-Lorentzian lineshape with on-resonance singularity handled via extrapolation (see appendix).

## 3. Methods

**Figure 3.**
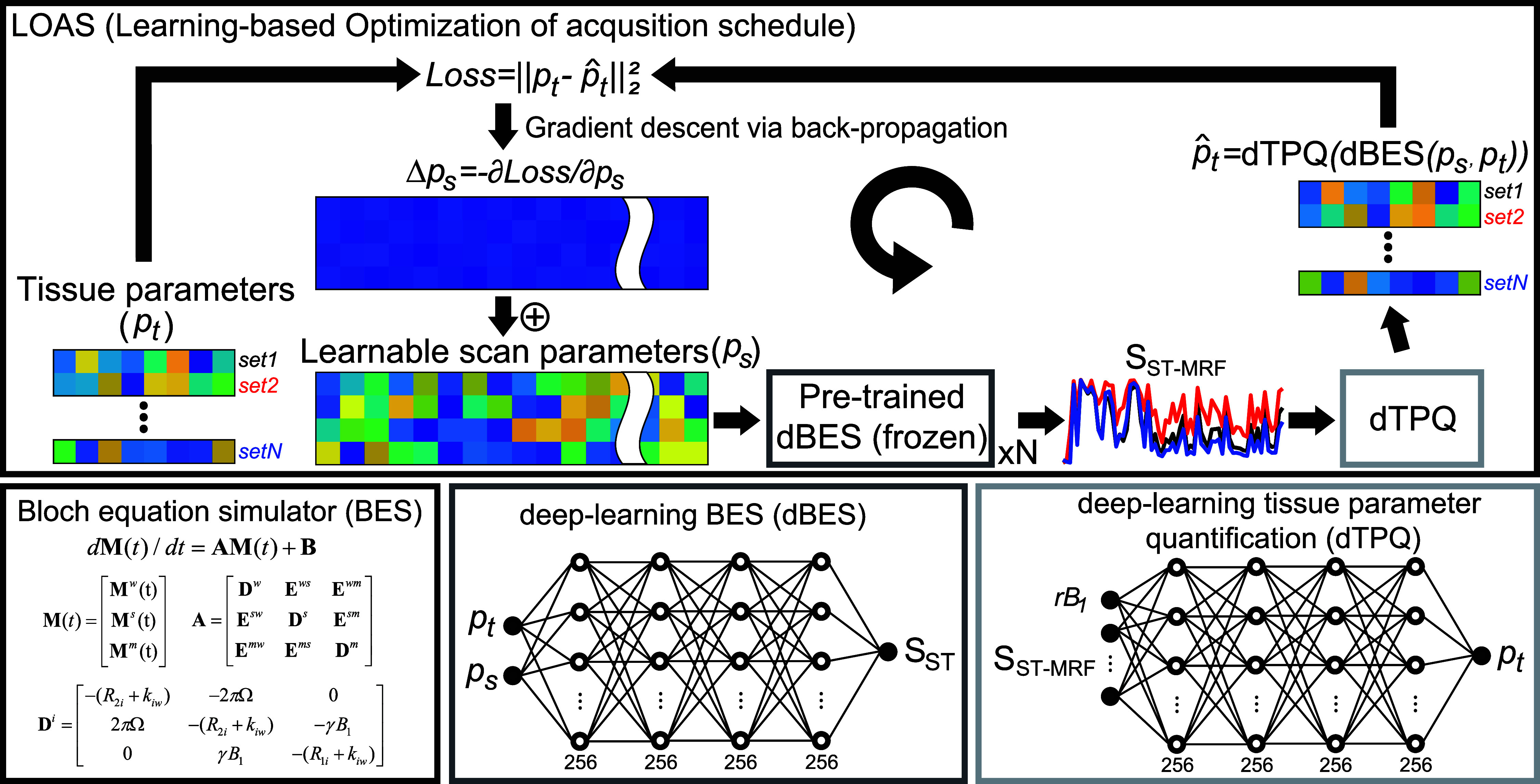
A schematic diagram of the proposed method. Two deep neural networks are incorporated in the MRF schedule optimization. Deep-learning Bloch equation simulator (dBES) is a surrogate forward model for the three-pool exchange model and deep-learning tissue parameter quantification network (dTPQ) is a network for tissue parameter estimation. The ST-MRF signals were synthesized from dBES with millions of tissue parameters and fed to dTPQ to estimate tissue parameters, which were then compared to the ground truth of the tissue parameters for a loss calculation. The calculated loss was back-propagated to simultaneously update the scan parameters and dTPQ with the Adam optimizer. Finally, the scan parameters were iteratively updated through epochs to approach the minimum of quantification errors. For visualization purposes, only four layers are illustrated, while the actual dBES and dTPQ networks comprise seven fully connected layers.

An overview of the LOAS tailored to ST-MRF is illustrated in figure 3. Here, we incorporate two deep neural networks into LOAS framework: (i) the deep-learning-based Bloch equation simulator (dBES), which is designed to solve the complex Bloch–McConnell equations with the three-pool exchange model as a surrogate forward model (parameter-to-signal) and (ii) the deep-learning-based tissue parameter quantification network (dTPQ), which efficiently solves the inverse Bloch equation problem (signal-to-parameter). During LOAS optimization, dTPQ is updated along with the MRI scan parameters (*p*_scan_) for estimating the best tissue parameter, while the dBES network is pre-trained and freezing for generating MRF signals based on given tissue and scan parameters in a deterministic manner. As a result, the proposed framework can efficiently find optimal solutions (MRF acquisition schedules) by minimizing quantification errors across multiple tissue parameters, even with non-differentiable signal model.

### 3.1. dBES

A fully-connected neural network (FCNN) architecture was designed to generate ST-MRF profiles by solving a forward problem involving nonlinear, non-differentiable Bloch–McConnell equations with a three-pool exchange model (figure 3). The network estimated ST-MRF profiles based on given tissue parameters and MRI scan parameters as follows:

\begin{equation*}{S_{{\mathrm{ST}} - {\mathrm{MRF}}}} = {\mathrm{dBES}}\left({p_{\mathrm{t}}},{p_{\mathrm{s}}}\right)\end{equation*}

\begin{equation*}{p_{\mathrm{t}}} = \left[T_1^{\mathrm{w}},T_2^{\mathrm{w}},{k_{{\mathrm{mw}}}},M_0^{\mathrm{m}},T_2^{\mathrm{m}},{\Delta _{{\mathrm{mw}}}},{k_{{\mathrm{sw}}}},M_0^{\mathrm{s}},T_2^{\mathrm{s}},\Delta {B_0},r{B_1}\right]\end{equation*}

\begin{equation*}{p_{\mathrm{s}}} = \left[{B_1},\Omega ,{T_{\mathrm{s}}},{T_{\mathrm{d}}}\right]\end{equation*}

where *S*_ST-MRF_ is the estimated ST-MRF signal, *p*_t_ represents the set of tissue parameters, and *p*_s_ represents the set of scan parameters. To enhance the speed and accuracy of training, both the scan and tissue parameters were normalized to a range of [0 1]. The dBES network comprised seven fully-connected layers with 256 channels each, followed by rectified linear units (ReLUs) as the activation function. The final layer used the sigmoid function to constrain the output signal range to between 0 and 1. Note that dBES produces the MRF signal based on a single set of tissue and scan parameters. For the training dataset, one hundred million sets of tissue parameters were sampled from the pre-defined range of each parameter (table 1). The network was implemented using Pytorch on an NVIDIA TITAN RTX GPU (Santa Clara, CA) and trained for 100 epochs using the adaptive moment estimation (Adam) optimizer (Kingma and Ba 2014) with a batch size of 256. The initial learning rate was 10^−4^ and decreased by a factor of 0.1 for every 20 epochs. The training dataset was randomly divided into two subsets: 90% for training and 10% for validation. The network exhibiting the lowest validation loss was selected for LOAS optimization. Bloch simulations (as ground-truth values for dBES training) were performed on a 64-bit Windows operating system (12-core, 3.8 GHz AMD Ryzen 9 3900XT processor and 32 GB of memory) using MATLAB (MathWorks, Natick, MA).

**Table 1.** The characteristics of tissue parameters used in the data preparation step (training dataset).

	Tissue parameters									
	*K*_sw_ (Hz)	*M*_0_^s^ (mM)	*k*_mw_ (Hz)	*M*_0_^m^ (M)	*T*_2_^m^ (*µ*s)	Δ_mw_	*T*_1_^w^ (s)	*T*_1_^w^/*T*_2_^w^	Δ*B*_0_ (Hz)	*rB*_1_
Upper bound	500	550	100	27.5	100.0	5	3.0	30.0	128	1.5
Lower bound	5	0	5	0	1.0	0	0.2	1.0	−128	0.5

### 3.2. dTPQ

Another FCNN architecture was developed to quantify multiple tissue parameters, including free bulk water, semisolid macromolecules, CEST, or solute protons, and ΔB_0_. In addition, the network was designed to perform dynamic scan-wise linear calibration of the RF saturation strength using measured *rB*_1_ values (Windschuh *et al* 2015, Kang *et al* 2023),

\begin{equation*}{p_{{{\mathrm{t}^{\prime}}}}} = {\mathrm{dTPQ(}}{S_{{\mathrm{ST}} - {\mathrm{MRF}}}},r{B_1})\end{equation*}

\begin{equation*}{p_{{{\mathrm{t}^{\prime}}}}} = \left[T_1^{\mathrm{w}},{k_{{\mathrm{mw}}}},M_0^{\mathrm{m}},T_2^{\mathrm{m}},{\Delta _{{\mathrm{mw}}}},{k_{{\mathrm{sw}}}},M_0^{\mathrm{s}},\Delta {B_0}\right]\end{equation*}

where *S*_ST-MRF_ represents the ST-MRF signals, *p*_t*’*_ denotes the set of estimated tissue parameters and Δ*B*_0_, and *rB*_1_ is the relative *B*_1_ value that describes *B*_1_ inhomogeneity. The tissue parameters were normalized to a range of [0 1]. The dTPQ network consists of seven fully connected layers with 256 channels each, followed by a ReLU activation function. For the training dataset, forty million sets of tissue parameters were sampled from the pre-defined range for each parameter (table 1). The network was implemented using Pytorch on an NVIDIA TITAN RTX GPU (Santa Clara, CA) and trained for 100 epochs using the Adam optimizer with a batch size of 256. The initial learning rate was 10^−4^ and was reduced by a factor of 0.1 every 30 epochs. Various levels of white Gaussian noise (SNR ranging from 40 to 46 dB) were randomly added to the simulated ST-MRF signals.

### 3.3. ST-MRF acquisition schedules for comparison studies

Several ST-MRF schedules, including linear, PR, and LOAS-based optimized acquisition schedules, were generated and evaluated to assess their reconstruction accuracy.

#### 3.3.1. Linear acquisition schedule

In the linear acquisition schedule of ST-MRF, each scan parameter was incrementally increased or decreased from the minimum value to the maximum value within its specified range. The parameter ranges were as follows: 0.5–2.4 *µ*T for *B*_1_; 0.3–2.5 s for *T*_s_; and 1.5–5.0 s for *T*_d_. The frequency offset range varied based on the target tissue parameters: 8–40 ppm and −8 to −40 ppm for MTC; 3–4 ppm for APT; and −1–1 ppm for direct water saturation and Δ*B*_0_. The negative frequency offsets far from the water resonance were also included to evaluate the MTC asymmetric effect. Different numbers of dynamic scans were allocated for each parameter: three dynamic scans for direct water and *B*_0_, 20 dynamic scans for MTC, and 40 dynamic scans for APT parameters. The ranges of scan parameter values, particularly for RF saturation-related parameters, were constrained by specific absorption rate (SAR) limits. Specifically, RF saturation strength and duration were kept within the clinically permissible SAR levels due to the SAR-intensive nature of pseudo-continuous RF saturation with a 100% duty cycle enabled by a pTX technique and 3D turbo spin echo (TSE) readout with multiple inversion pulses (Heo *et al* 2019b). The same parameter ranges and SAR constraints were applied to other ST-MRF schedules.

#### 3.3.2. PR acquisition schedule

The PR acquisition schedule aimed to minimize data redundancy across multiple dynamic scans. For example, it included combinations such as high *B*_1_ with long *T*_s_, high *B*_1_ with short *T*_s_, low *B*_1_ with long *T*_s_, and low *B*_1_ with short *T*_s_. These combinations were chosen to ensure the lowest redundancy between scans. A set of scan parameters was then randomly selected based on these criteria. The SAR constraint was enforced by excluding scan parameters that did not meet the SAR limits during the random selection process.

#### 3.3.3. LOAS

The LOAS framework, integrated with the dBES and dTPQ networks, optimizes an MRF acquisition schedule to enhance the accuracy of tissue parameter quantification. During the training of the LOAS framework, ST-MRF signal profiles are synthesized using the dBES network. Subsequently, multiple tissue parameters are estimated from the dTPQ network, with the synthesized ST-MRF profiles and the relative B_1_ value as inputs. These estimates are then compared to the ground-truth tissue parameters to compute the loss. The calculated loss is back-propagated to simultaneously update the scan parameters and dTPQ using the Adam optimizer. The scan parameters are iteratively updated through epochs to approach the minimum of quantification errors. The estimated loss (*L*_t_) can be described with the *l*_1_-norms as follows:

\begin{equation*}{L_{\mathrm{t}}} = { }|{p_{\mathrm{t}}} - {\mathrm{dTPQ}}\left({\mathrm{dBES}}\left({p_{\mathrm{t}}},{p_{\mathrm{s}}};\eta \right),r{B_1}\right){|_1}\end{equation*}

where *η* represents the noise. The SAR constraint is incorporated as an additional loss as follows:

\begin{equation*}{L_{{\mathrm{total}}}} = { }{L_{\mathrm{t}}} + |{\mathrm{max}}\left({B_1} \times {T_{\mathrm{s}}} - {\mathrm{SAR}}\_{\mathrm{bound,}}0\right){|_1}\end{equation*}

where SAR_bound denotes the upper limit of the SAR constraint, set empirically to 4, which is the product of RF saturation strength and duration. For the training dataset, one hundred million sets of tissue parameters were randomly selected within a pre-defined range for each parameter (table 1). The range of *B*_0_ inhomogeneity (Δ*B*_0_) was limited to −64 to 64 Hz for the LOAS training, while a range of −128 Hz to 128 Hz was used for dTPQ training. The network was implemented using Pytorch on an NVIDIA TITAN RTX GPU (Santa Clara, CA). It was trained for 100 epochs with the Adam optimizer and a batch size of 256. The initial learning rate was 10^−2^, decreased by a factor of 0.1 every 60 epochs for scan parameters. For dTPQ, the initial learning rate was 10^−4^, with a decrease by a factor of 0.1 every 30 epochs. Because the scan parameters and the dTPQ network were updated simultaneously, different hyper-parameters were applied. The training dataset was randomly divided into two parts: 90% for training and 10% for validation. The MRF schedule with the lowest validation loss was selected as the optimal schedule. White Gaussian noise at various levels (SNR of 40–46 dB) was randomly added to the simulated ST-MRF signals.

### 3.4. Bloch–McConnell simulation studies

The ST-MRF signals were simulated using three-pool Bloch–McConnell equations to evaluate the performance of the deep-neural-network-based MRF signal synthesis (dBES) and dTPQ with various MRF acquisition schedules.

#### 3.4.1. Digital phantom simulations for dBES

Two simulation studies were conducted to assess the accuracy of ST-MRF signal synthesis using different combinations of tissue and scan parameters. In the first study, only the exchange rate (*k*_sw_) and concentration (*M*_0_^s^) of amide protons were varied, while other tissue parameters were kept fixed (*T*_1_^w^ = 1 s; *T*_2_^w^ = 1 s;). The scan parameters were set at 1.5 *μ*T for *B*_1_, 3.5 ppm for Ω, 2 s for *T*_s_, and 5 s for *T*_d_. In the second study, ten thousand sets of tissue parameters were randomly selected within the predefined ranges. ST-MRF profiles were synthesized using 1000 different MRF schedules, each consisting of 63 dynamic scans. The ST-MRF signals synthesized by dBES were compared to those obtained from conventional Bloch–McConnell equation simulator (BES).

#### 3.4.2. Digital phantom simulations for LOAS and dTPQ

To assess the efficacy of optimized MRF schedules, eight digital phantoms were constructed using Bloch–McConnell simulations. Each phantom contained five circular compartments to evaluate each of the eight tissue parameters, while the remaining seven tissue parameters were randomly selected from the predefined ranges (table 1) for all five circular compartments. The five uniform values for each tissue parameter in each phantom were as follows: 50, 150, 250, 350, and 500 Hz for *k*_sw_; 1, 125, 250, 375, and 500 mM for *M*_0_^s^; 5, 25, 50, 75, and 100 Hz for *k*_mw_; 5, 10, 15, 20, and 25 M for *M*_0_^m^; 1, 25, 50, 75, and 100 *μ*s for *T*_2_^m^; 1, 2, 3, 4, and 5 ppm for *Δ*_mw_; 0.2, 0.9, 1.6, 2.3, and 3.0 s for *T*_1_^w^; and −80, −40, 0, 40, and 80 Hz for *ΔB*_0_. Using the ground-truth tissue parameters, ST-MRF images were generated through dBES and subsequently processed with the dTPQ network to estimate tissue parameters (also see supplementary figure S1). The dTPQ model was evaluated using normalized root mean square errors (nRMSEs) and mean absolute errors (MAEs) as metrics for predictive accuracy.

In addition, digital phantom studies were performed to assess the precision of tissue parameter estimations, specially focusing on the ability to discriminate small differences in quantitative values, particularly for the exchange rate and concentration of amide protons. The amide proton exchange rates ranged from 50 Hz to 500 Hz in 10 Hz increments, while the other tissue parameters were randomly selected from the predefined ranges. Similarly, amide proton concentrations varied from 10 mm to 500 mm in 10 mm increments. The tissue parameter maps estimated by dTPQ were compared to the ground-truth values. Adjacent compartments were statistically compared by using a two-sided Student’s t-test, with statistical significance set at *p* < 0.05.

### 3.5. *In vivo* MRI experiments

Four healthy volunteers (two males and two females; age: 22–29) and one brain tumor patient (a 62 year-old male with IDH-mutant oligodendroglioma, MGMT methylation, and 1p/19q codeletion, Grade 2) were scanned on a 3 T MRI system (Achieva dStream, Philips Healthcare, Best, the Netherlands). The human studies were approved by the Johns Hopkins Institutional Review Board. The 3D ST-MRF images were acquired using a fat-suppressed (spectral pre-saturation with inversion recovery), multi-shot TSE pulse sequence with 2 × 2 compressed sensing accelerations in the two phase-encoding directions (*k_y_*–*k_z_*) (Heo *et al* 2017c). Imaging parameters included TE = 6 ms; FOV = 212 × 186 × 60 mm^3^; spatial resolution = 1.8 × 1.8 × 4 mm^3^; slice-selective 120° refocusing pulses; turbo factor = 104; and slice oversampling factor = 1.4. Each subject was scanned using the LOAS, PR and linear schedules, each comprising 63 dynamic scans. Pre-processing included image registration, signal normalization, and denoising for all schedules. For signal normalization, an additional unsaturated image (*S*_0_) was acquired. To compensate for motion artifacts, all dynamic scans were registered to the *S*_0_ image using a spatial transformer network (STN) with a normalized cross-correlation (NCC) loss (Lee *et al* 2023). The STN was optimized individually for each subject to estimate a 2D affine transformation matrix. The network was optimized for 200 epochs with a learning rate of 10^−4^ and a batch size of one. To enhance ST-MRF reconstruction, the MD-S2S (multidimensional-self2self) method, which is a recently proposed self-supervised denoising technique (Kang *et al* 2024), was applied to the normalized ST-MRF images. Due to the lack of a training dataset, we adopted an optimization scheme for denoising where train and test datasets were the same. The network was optimized for 10 000 epochs with a learning rate of 10^−4^ and a batch size of one. For a comparison study, *B*_0_ mapping was performed using the water saturation shift referencing (WASSR) method with the following parameters: 26 frequency offsets (from −1.5 to 1.5 ppm at intervals of 0.125 ppm); *T*_s_ = 800 ms; and *B*_1_ = 0.5 *μ*T. For *B*_1_ mapping, the dual refocusing echo acquisition mode (DREAM) method was used with the simulated echo acquisition mode (STEAM) with a flip angle of 40° (Nehrke and Bornert 2012). Regions of interests (ROIs) were carefully drawn on *T*_2_ maps (see supplementary figure S2). The between-subject coefficient of variation (CoV) was computed as the ratio of the standard deviation to the mean across all subjects, expressed as a percentage. A CoV of less than 15% was considered indicative of excellent repeatability of the tissue parameters.

#### 3.5.1. Validation of ST-MRF using synthetic MRI

Validating the proposed multipool tissue parameter estimation is challenging due to the lack of a reliable *in vivo* ground truth. To enable effective validation, a synthetic MRI analysis was performed using *in vivo* images to assess the proposed optimal ST-MRF method. Tissue parameters estimated from 63 ST-MRF images acquired with the LOAS schedule were used to synthesize new ST-MRF images based on the new schedule with 20 dynamic scans. In addition, experimentally obtained water T_2_ maps were incorporated into the image synthesis. The synthetic ST-MRF images were then compared with the experimentally acquired images obtained under the same MRI acquisition settings by calculating the root mean square error (RMSE).

## 4. Results

### 4.1. *Bloch*–*McConnell simulation studies*

#### 4.1.1. dBES

**Figure 4.**
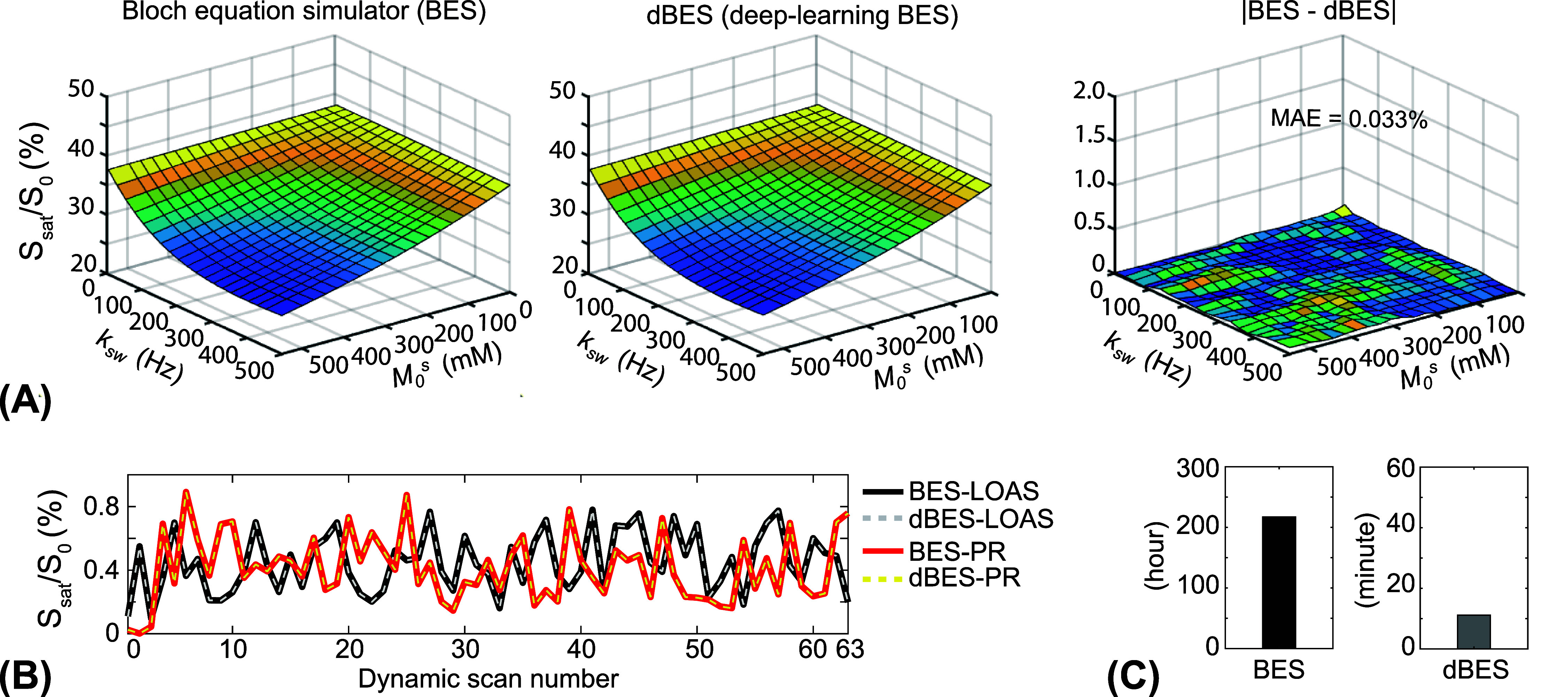
Bloch–McConnell equation-based simulations. (A) The ST-MRF signals were generated from the dBES, with varying exchange rates and concentrations of amide protons, compared to reference signals obtained from the conventional Bloch–McConnell equation simulator (BES). The difference in the synthesized signals is also shown. (B) Two schedules of LOAS and PR with 63 dynamic scans were used to generate ST-MF signals using BES and dBES. (C) The comparison for computation cost of MRF signal synthesis with 40 million sets of tissue parameters.

The accuracy of dBES for MRF signal synthesis was evaluated using Bloch simulations based on the three-pool exchange model. Figure 4(A) illustrates the MRF signals synthesized by dBES, with varying exchange rates and concentrations of amide protons, compared to reference signals obtained from the conventional BES. The synthesized signals from dBES showed excellent agreement with those from the BES, with MAE of 0.036%. It is evident that higher exchange rates and concentrations generate smaller signal intensities. The performance of dBES was further assessed with various MRF schedules. Ten thousand schedules, each with 63 dynamic scans, were used to synthesize ST-MRF signals. The synthesized ST-MRF signals from dBES were in excellent agreement with the reference signals from BES across ten thousand sets of tissue parameters. The MAE values for the 1000 schedules were 0.051%. Figure 4(B) shows two representative signals generated by dBES and BES using the LOAS and PR schedules. In addition, the computational cost of dBES was compared to that of the BES by measuring computation time required for ST-MRF signal synthesis using forty million sets of tissue parameters and the LOAS schedule with 63 dynamic scans (figure 4(C)). While the Bloch simulator required approximately 220 h, dBES completed the task in about 10 min, achieving a dramatic reduction in synthesis time by approximately 1000-fold.

#### 4.1.2. LOAS and dTPQ

**Figure 5.**
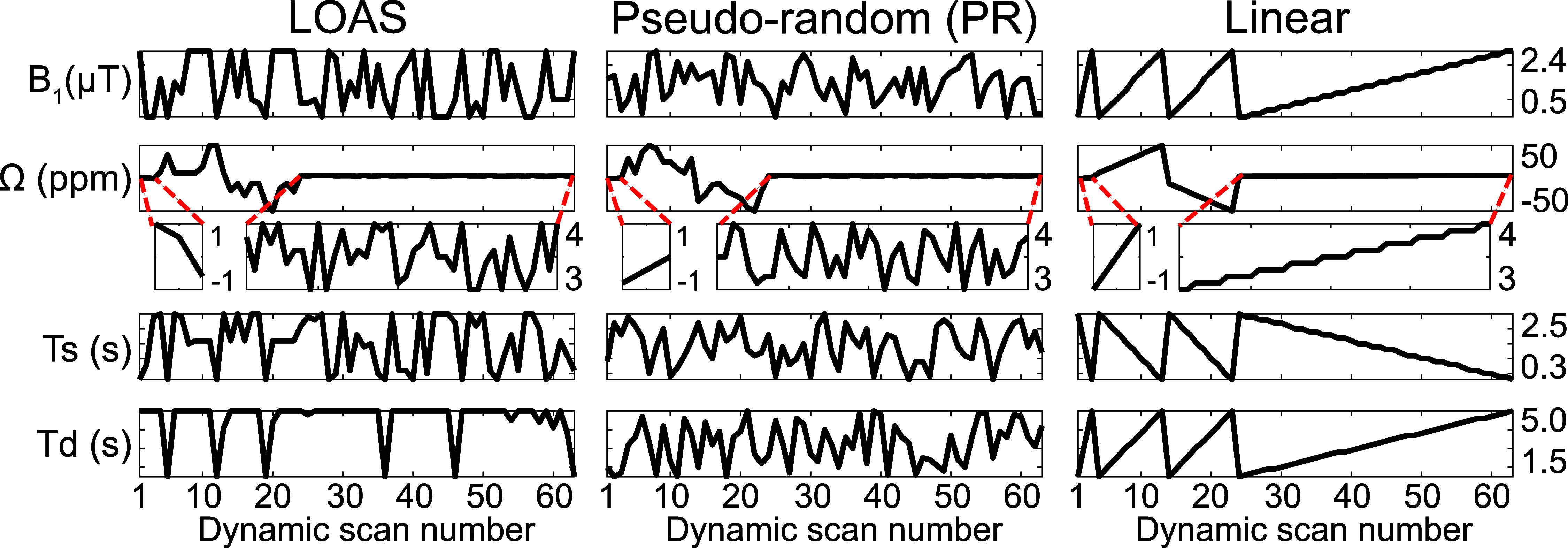
Optimized ST-MRF acquisition schedules (*B*_1_, Ω, *T*_s_, and *T*_d_) with 63 dynamic scans from the LOAS algorithm compared to the pseudo-random (PR) and linear schedules.

**Figure 6.**
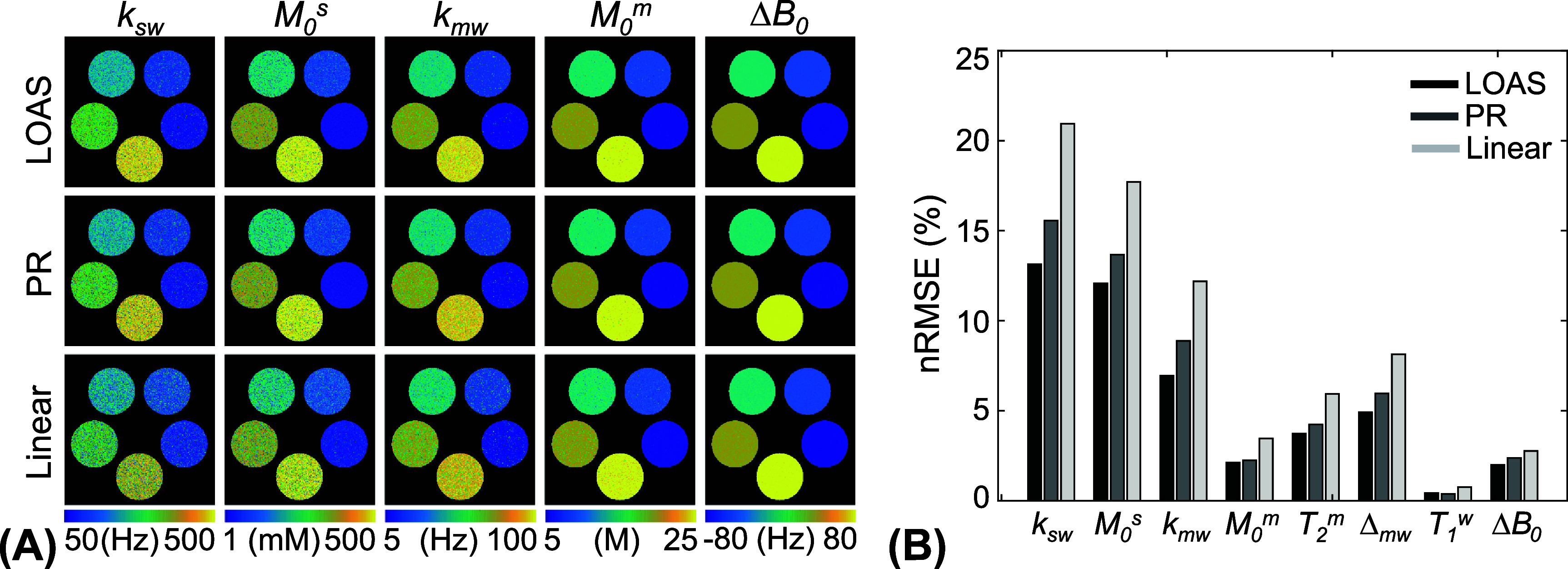
Bloch-equation-based digital phantom study with an SNR of 46 dB. (A) Tissue parameter maps were estimated by dTPQ with various ST-MRF schedules (LOAS, PR, and linear). (B) The normalized root mean square error (nRMSE) was calculated by comparing the estimated parameters and the ground truths for the three schedules.

The ST-MRF acquisition schedule was optimized using the LOAS algorithm and compared with the PR and linear schedules (figure 5). The quantification accuracy was assessed by calculating the nRMSE and MAE metrics between tissue parameter estimates obtained with the different MRF schedules and the ground-truth values, as shown in figure 6. The average nRMSE values of LOAS, PR, and linear schedules were as follows: 13.1%, 15.6%, and 20.1% for *k*_sw_; 12.1%, 13.7%, and 17.7% for *M*_0_^s^; 7.0%, 8.9%, and 12.2% for *k*_mw_; 2.1%, 2.2%, and 3.5% for *M*_0_^m^; 3.8%, 4.2%, and 5.9% for *T*_2_^m^; 4.8%, 5.7%, and 8.4% for Δ_mw_; 0.44%, 0.39%, and 0.77% for *T*_1_^w^; and 2.0%, 2.4%, and 2.8% for Δ*B*_0_, respectively. The average MAE values for the LOAS, PR, and linear schedules were: 36.6 Hz, 46.9 Hz, and 73.1 Hz for *k*_sw_; 27.0 mm, 33.6 mm and 54.1 mm for *M*_0_^s^; 3.2 Hz, 4.8 Hz, and 7.2 Hz for *k*_mw_; 0.22 M, 0.26 M and 0.50 M for *M*_0_^m^; 0.9 *μ*s, 1.1 *μ*s, and 2.1 *μ*s for *T*_2_^m^; 0.10 ppm, 0.12 ppm, and 0.21 ppm for Δ_mw_; 7.3 ms, 7.8 ms, and 15.1 ms for *T*_1_^w^; and 1.0 Hz, 1.3 Hz, and 1.5 Hz for Δ*B*_0_, respectively. Overall, the LOAS schedule yielded lower nRMSE and MAE values compared to the PR and linear schedule. This trend was consistent with the higher noise level of an SNR of 40 dB (see supplementary figure S3).

**Figure 7.**
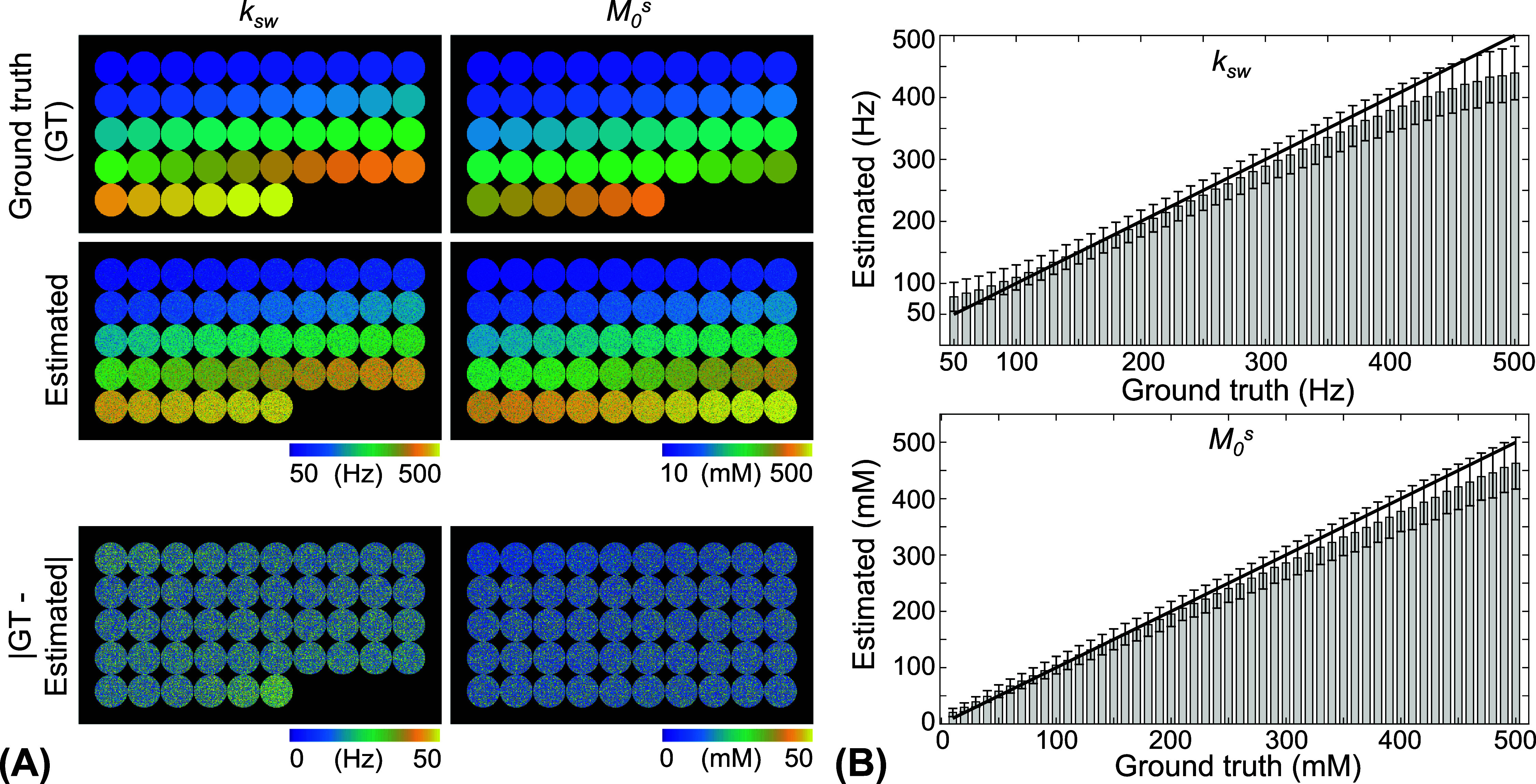
Bloch-equation-based digital phantom study to assess the precision of tissue quantification with an SNR of 46 dB. (A) The quantitative parameter maps estimated from the LOAS schedule were compared to the ground truth. (B) The estimated values of each compartment were compared to the ground truth values for the exchange rate and concentration of the amide proton.

In addition, the resolution of tissue parameter quantification was evaluated using numerical phantoms with multiple compartments, each parameterized with small step sizes, specifically 10 Hz for the amide proton exchange rate and 10 mm for the concentration. As shown in figure 7, the quantitative tissue parameter maps estimated using the LOAS schedule were comparable to the ground-truth values. Furthermore, the estimated values for each compartment did not significantly differ from the ground-truth values and were statistically different from those of adjacent compartments for nearly every tissue parameter, with the exception of compartments at 480 and 490 Hz. This finding was also valid under higher noise conditions with an SNR of 40 dB (supplementary figure S4).

### 4.2. *In vivo* experiments

**Figure 8.**
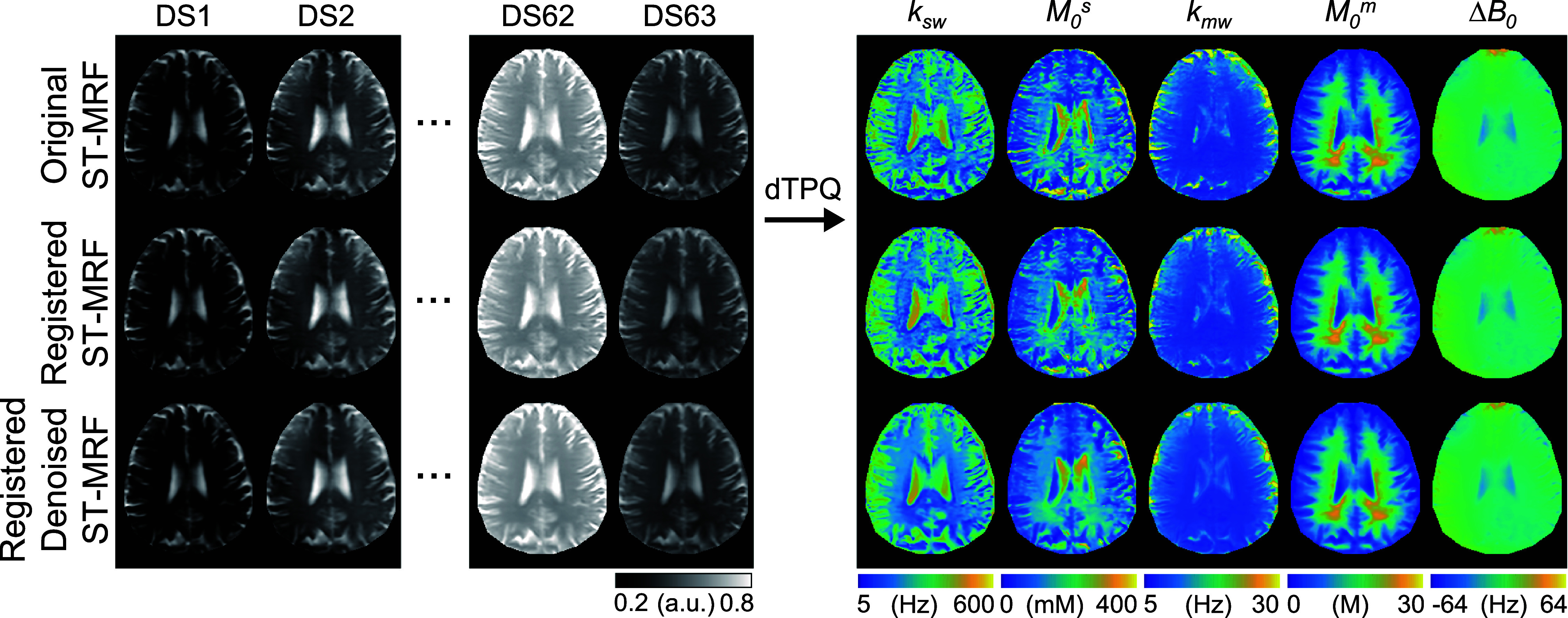
*In vivo* tissue parameter maps of a representative healthy volunteer, estimated from the original ST-MRF images, are compared to those obtained from registered (motion-corrected) ST-MRF images and from registered and denoised ST-MRF images using the LOAS schedule.

**Figure 9.**
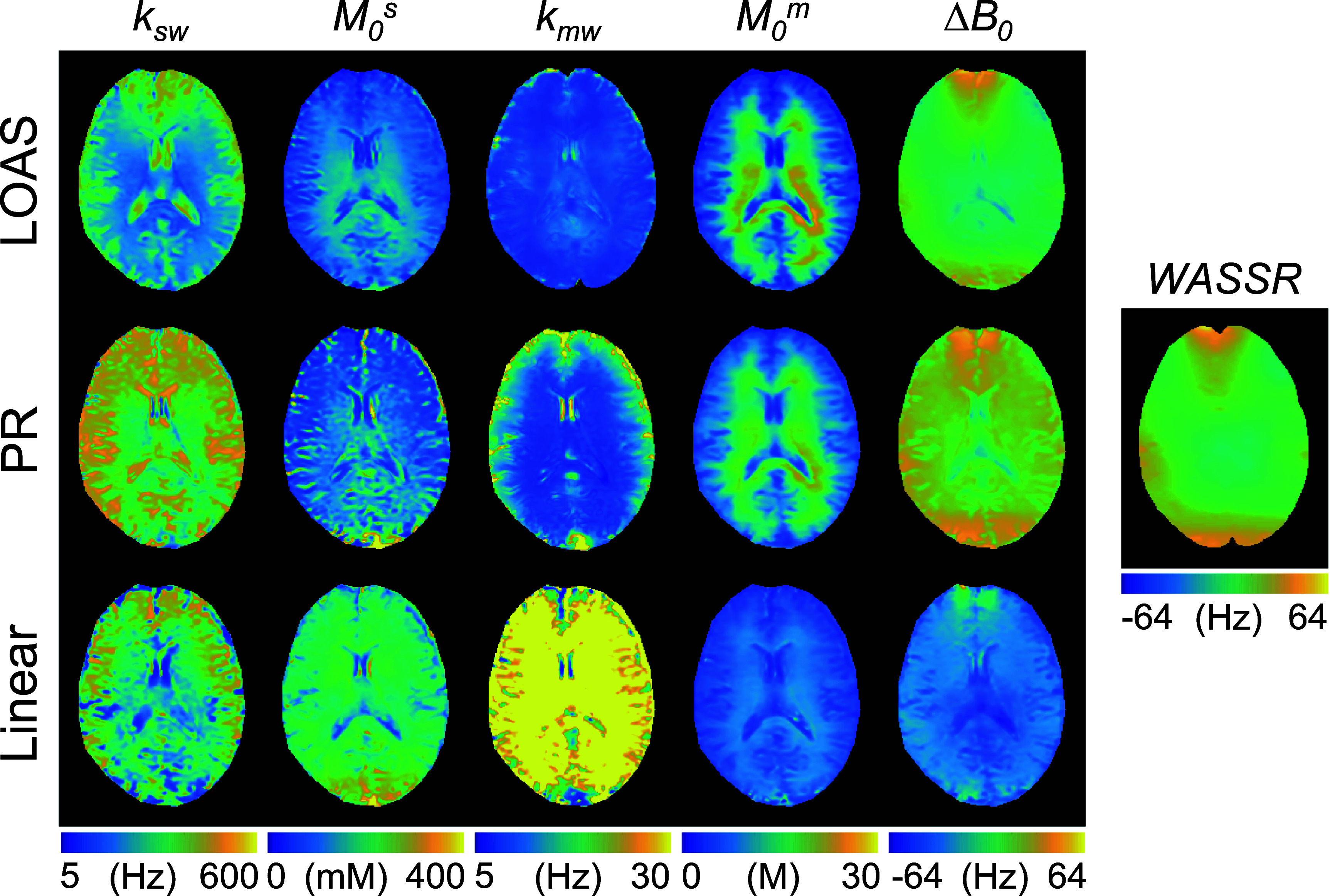
*In vivo* tissue parameter maps of a representative human brain of a healthy volunteer estimated from the LOAS, PR, and linear schedules.

ST-MRF images acquired from healthy human brains were enhanced by reducing motion artifacts using the STN network and suppressing noise with the MD-S2S denoising method (figure 8). The resulting registered and denoised ST-MRF images, obtained using the optimized LOAS, PR, and linear acquisition schedules, were then used to estimate tissue parameter maps using the dTPQ networks respectively trained for each schedule (figure 9). Importantly, the acquired *rB*_1_ map from the DREAM method was used to correct the B_1_ inhomogeneity in the dTPQ network. The exchange rate and the concentration maps of the semisolid macromolecular protons were relatively consistent between the LOAS and PR schedules, whereas the linear schedule did not provide reliable tissue parameter maps. The exchange rate and the concentration maps of the amide protons also varied significantly among the different acquisition schedules. Table 2 presents the quantitative parameter values estimated from the LOAS schedule for white matter (WM). The amide proton exchange rate for gray and WM were approximately 285 Hz (95% confidence interval [CI]: 199–370 Hz) and 221 Hz (95% CI: 144–299 Hz), respectively, and the concentration for gray and WM were approximately 111 mM (95% CI: 86–136 Hz) and 148 mM (95% CI: 97–198 Hz). Furthermore, the semisolid macromolecular proton exchange rates rate for gray and WM were approximately 12.2 Hz (95% CI: 7.2–17.2 Hz) and 9.2 Hz (95% CI: 7.7–10.6 Hz), respectively, and the concentration for gray and WM were approximately 11.9 M (95% CI: 9.1–14.8 M) and 19.9 M (95% CI: 13.8–26.0 M), respectively. Between-subject CoV values of less than 15% were observed for most parameters, demonstrating the high reliability of the proposed quantification. The Δ*B*_0_ map estimated from the proposed network with the LOAS schedule was in excellent agreement with the Δ*B*_0_ map estimated using the WASSR method. In contrast, the Δ*B*_0_ maps estimated from the PR and linear schedules showed poor agreement with the reference WASSR map. The MAE values between the estimated Δ*B*_0_ and WASSR maps were 5.56 Hz for LOAS, 8.39 Hz for PR, and 41.09 Hz for linear schedules.

**Table 2.** Estimated tissue parameters for gray matter (GM) and white matter (WM) from the registered and denoised ST-MRF images of the healthy volunteer human brains (*n* = 4).

	*K*_sw_ (Hz)		*M*_0_^s^ (mM)		*K*_mw_ (Hz)		*M*_0_^m^ (M)		*T*_2_^m^ (*µ*s)		Δ_mw_ (ppm)		*T*_1_^w^ (s)	
	GM	WM	GM	WM	GM	WM	GM	WM	GM	WM	GM	WM	GM	WM
1	264	232	113	156	10.1	9.2	13.0	18.9	15.4	15.2	1.00	1.27	1.44	1.27
2	306	214	100	125	12.6	9.5	12.3	18.3	14.7	15.4	0.84	0.99	1.37	1.28
3	259	191	119	160	13.8	8.5	10.9	22.6	15.1	15.4	1.21	1.23	1.41	1.24
4	310	248	112	149	12.2	9.4	11.6	19.7	15.2	15.5	1.14	1.38	1.38	1.22

Mean ± SD	285 ± 27	221 ± 24	111 ± 8	148 ± 16	12.2 ± 1.6	9.2 ± 0.4	11.9 ± 0.9	19.9 ± 1.9	15.1 ± 0.3	15.4 ± 0.1	1.05 ± 0.16	1.23 ± 0.17	1.40 ± 0.03	1.25 ± 0.03

95% CI	199–370	144–299	86–136	97–198	7.2–17.2	7.7–10.6	9.1–14.8	13.8–26.0	14.2–16.0	15.0–15.4	0.53–1.56	0.70–1.77	1.30–1.50	1.17–1.34

p-value	0.01		<0.01		0.01		<0.001		NS		NS		<0.001	

CoV (%)	9.4	11.0	7.1	10.7	12.9	4.8	7.5	9.6	1.9	0.8	15.5	13.7	2.2	2.1

**Figure 10.**
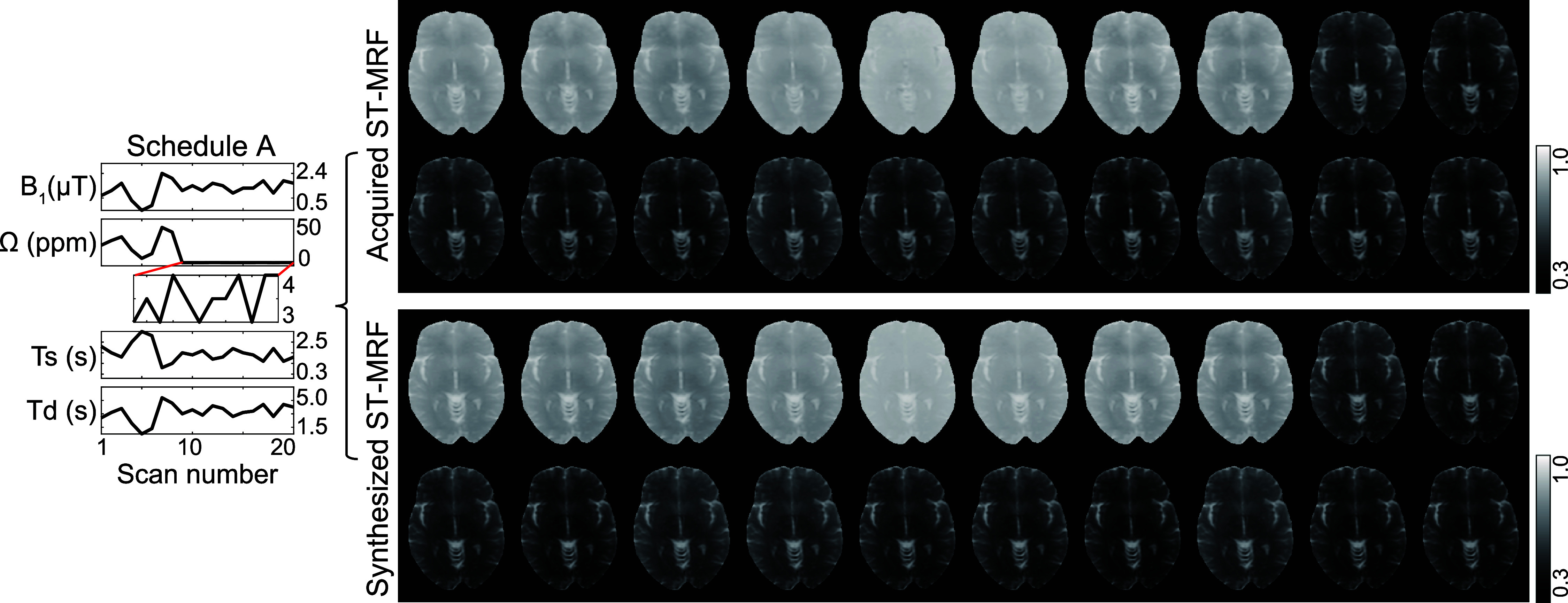
Synthetic MRI analysis for validating estimated *in vivo* tissue parameter maps. The *in vivo* tissue parameter maps estimated using the dTPQ (Deep-learning tissue parameter quantification network) with the LOAS schedule were fed to Bloch equation simulator (BES) to synthesize ST-MRF images based on Schedule A. The resulting synthetic images were then compared to experimentally acquired ST-MRF images.

**Figure 11.**
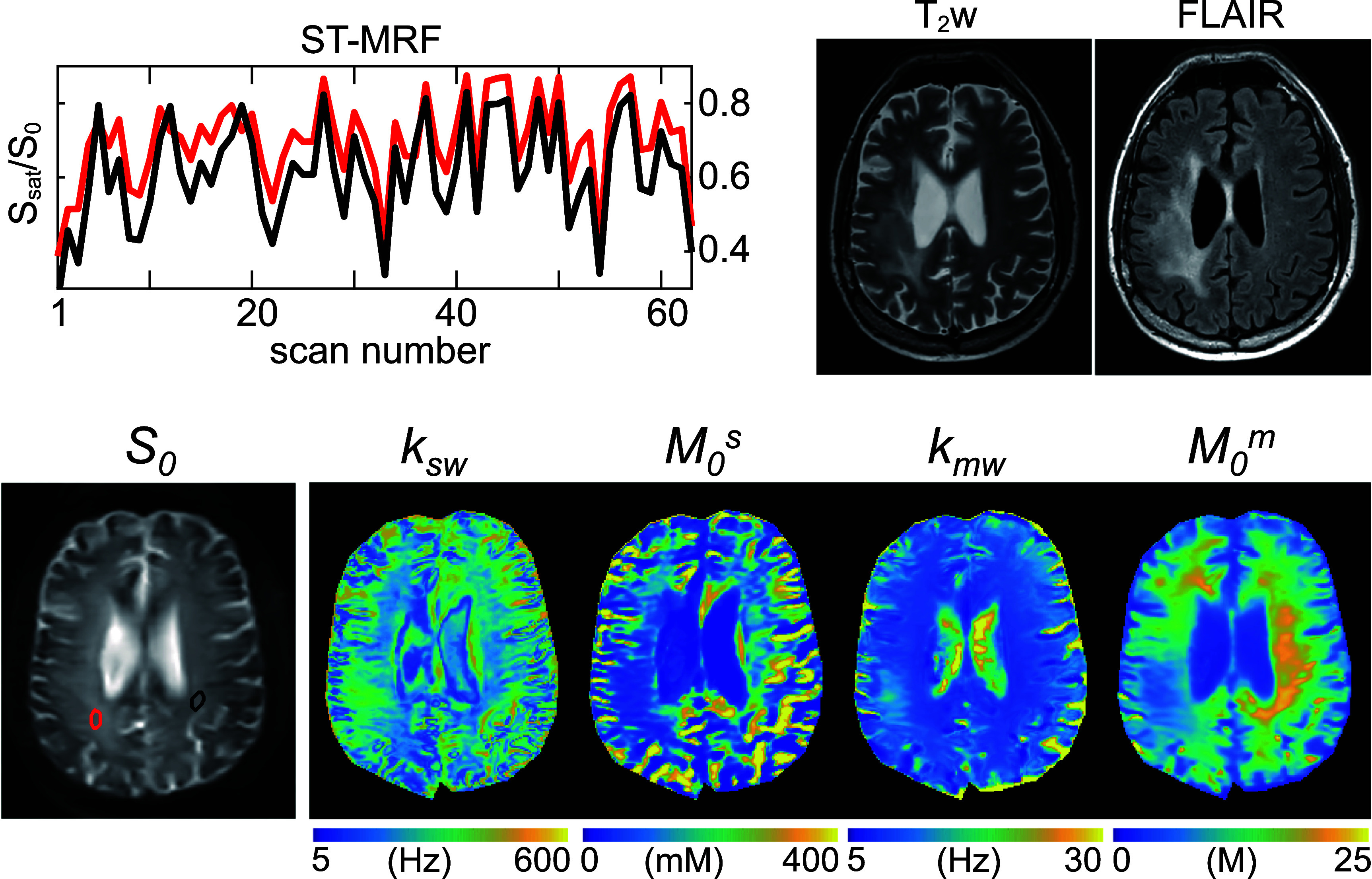
Representative ST-MRF signal evolutions across 63 dynamic scans are shown for the tumor region (red) and the contralateral region (black) in a brain tumor patient with oligodendroglioma. Each ROI is carefully drawn on the corresponding normalization scan (*S*_0_). The estimated quantitative tissue-parameter maps are displayed alongside corresponding *T*_2_-weighted (*T*_2_w) and fluid-attenuated inversion-recovery (FLAIR) images.

To validate the proposed method, ST-MRF images were synthesized and compared with experimental measurements due to lack of reliable *in vivo* ground truth. The quantitative tissue parameter maps, estimated from 63 LOAS ST-MRF images using dTPQ, were input to BES to generate synthetic ST-MRF images corresponding to a new, previously unseen acquisition schedule (See supplementary figure S5). As shown in figure 10, the synthesized images demonstrated a high degree of agreement with experimentally acquired ST-MRF images using this new schedule, with an average RMSE of 0.0118. Figure 11 presents the ST-MRF profiles and reconstructed tissue-parameter maps derived from optimized MRF schedule in a brain tumor patient with oligodendroglioma. Interestingly, the *k*_sw_ and *k*_mw_ values were slightly higher in the *T*_2_w and fluid-attenuated inversion-recovery (FLAIR) hyperintense regions, where no Gd enhancement was observed (data not shown). In contrast, the *M*_0_^s^ and *M*_0_^m^ values showed a reduction in these regions compared to the contralateral side.

## 5. Discussion

A deep Bloch equation simulator-based physics-informed optimization framework was developed to optimize acquisition schedules for non-differentiable signal models. This method utilized deep neural networks for efficient MRF profile synthesis and accurate quantification of tissue parameters in the human brain. In a previous LOAS framework, the Bloch equation simulator was directly integrated into the physics-informed optimization architecture to compute MRF signal profiles based on the current scan parameters. The gradient computed from the quantification loss with respect to the scan parameter was then used to update the scan parameters towards minimizing the loss. Thus, this approach is not applicable to non-differentiable model. Moreover, unlike the previous study, which used a two-pool exchange model to characterize the properties of water and semisolid macromolecule pools, we extended the model to a three-pool formulation incorporating the extrapolated super-Lorentzian RF absorption rate for the semisolid macromolecule pool. Typically, in biological tissue, the semisolid MT spectrum is better characterized by a super-Lorentzian lineshape, particularly when proton motion is restricted within proteins and myelin lipid bilayers (Morrison and Henkelman 1995, Quesson *et al* 1998, Singh *et al* 2026).

Modeling the semisolid MTC pool with a super-Lorentzian lineshape requires averaging dipolar interactions across all molecular orientations, which significantly increases the computational burden and necessitates extrapolation to address the on-resonance singularity. In our study, this complexity is alleviated through the use of a deep Bloch equation simulator. Consequently, the deep Bloch equation simulator used in this study not only circumvents the non-differentiability of signal model during gradient computation for backpropagation, but also enables rapid synthesis of the database. Traditional Bloch simulations required 220 h to generate 40 million training datasets for each MRF schedule, whereas the learning-based Bloch simulator achieved this in just 10 min. Although the learning-based Bloch simulator significantly reduced the time required for database construction, further acceleration is possible by eliminating repetitive training through the only-train-once MRF method (Kang *et al* 2022a). In addition, the ablation studies indicate that the performance of dBES is relatively robust to moderate variations in network architecture, with deeper networks and larger channel capacities generally achieving lower MAE (supplementary table S1). All tested models, however, maintained signal MAE below 0.1%. Moreover, a reduction in the training dataset size consistently leads to a degradation in accuracy, underscoring the critical role of large-scale training data. (supplementary table S2). Nonetheless, more advanced architectures, such as Kolmogorov–Arnold Networks (Liu *et al* 2025, Wang *et al* 2025) or implicit neural representations (Sitzmann *et al* 2020, Zhu *et al* 2025), could potentially improve performance. A systematic exploration of these advanced models is an important direction for future research.

To maximize the efficiency of sequence optimization, the unique characteristics of each proton pool were considered. Dynamic images were acquired around the water resonance frequency to capture *B*_0_ inhomogeneity effects, which allowed for the estimation of the Δ*B*_0_ map. To detect signals from amide protons centered at 3.5 ppm, the frequency offset ranged from 3 ppm to 4 ppm due to the relatively broad frequency profile of amide protons. In addition, data acquisitions within these frequency ranges were interpolated and shifted to compensate for the *B*_0_ inhomogeneity effect. In contrast, with a fixed frequency offset of 3.5 ppm, the error of amide proton parameter quantification increased significantly with B_0_ error, despite incorporating *B*_0_ inhomogeneity in the training of the dTPQ network (supplementary figure S6). Far off-resonance positive and negative frequency offsets were equally sampled to quantify the semisolid MTC parameters, including the asymmetric MTC effect. Overall, MTC parameters were accurately estimated even with a four-fold reduction in the number of dynamic scans: 20 in our study compared to 80 in the previous study including positive and negative frequency offsets (Singh *et al* 2023). Evaluating convergence stability and potential overfitting enhances the reliability of the proposed framework. To assess these factors, we performed the LOAS optimization multiple times using different random seeds and examined the resulting variability. As summarized in supplementary table S3, the proposed framework consistently converged to an optimal schedule that minimized quantification error across runs, demonstrating stable convergence behavior. In addition, we monitored the training, validation, and test losses during the training of dBES and dTPQ, as well as during LOAS optimization, and found no evidence of overfitting.

The apparent amide proton exchange rates and concentration of WM estimated using the proposed method with the LOAS schedule were approximately 233 Hz and 160 mm, respectively. These values differ somewhat from those reported in previous studies. Interestingly, water-exchange spectroscopy (WEX)-based methods reported much lower exchange rate and concentrations, likely because they utilized finite saturation pulse duration (several milliseconds) during the water labeling (Zhou *et al* 2003, 2004). However, amide protons are known to exhibit a wide range of exchange rates, including fast exchange rates at a physiologically relevant pH range (Wüthrich 1986). In addition, the WEX-based approaches estimated the exchange rate from the mixing-time-dependent signal evolution using a simplified model (APT ratio) that neglects MTC and direct water-saturation effects. While the estimated slow exchange rates are consistent with recent deep-learning-based CEST-MRF studies (Perlman *et al* 2022a, 2022b, Cohen and Otazo 2023), the training data used in those studies were limited to amide proton exchange rates below 100 Hz, which may inherently restrict estimation accuracy for faster-exchanging components. In contrast, our method employed a range of saturation powers and saturation times to encompass a broad spectrum of amide proton exchange rates and utilized a more comprehensive three-pool model that incorporates a super-Lorentzian lineshape to more accurately characterize *in vivo* brain tissue. Accordingly, discrepancies in the quantified amide proton parameters are likely driven by differences in training data sets, model assumptions, and MRF acquisition protocols. In addition, a fast exchange rate of amide protons was observed in the human study for voxels located within the cerebral ventricles. However, negligible APT signals are expected in the cerebrospinal fluid (CSF), suggesting that these erroneous values may be due to partial volume effects of CSF flow-related artifacts. On the other hand, the estimated values of the semisolid macromolecular proton parameters were consistent with previous quantitative MTC studies (Dortch *et al* 2011, Kim *et al* 2020, Kang *et al* 2021).

The validation of quantitative tissue parameters, particularly proton exchange rate and solute concentration, is essential for evaluating the reliability of imaging method and advancing the clinical translation of ST-MRF. However, a gold standard for *in vivo* tissue parameter measurement does not currently exist. While CEST physical phantoms can simulate simple biological tissues, they fall short of replicating the full complexity of *in vivo* tissue. In this study, synthetic MRI analysis was performed to indirectly validate ST-MRF method. Synthetic ST images were generated using various combinations of scan parameters (*B*_1_, *T*_s_, Ω, and *T*_d_) and compared to actual MR image acquisitions. The synthesized images showed good agreement with the corresponding acquisitions, indicating the reliability of the estimated parameters. Notably, the deep-learning-based quantification is robust to noise due to the inclusion of Gaussian noise in the simulated signals during training. Consequently, the synthesized ST images appear smoother than the experimentally acquired images.

Motion between image acquisitions can misalign a series of weighted images in quantitative MRI, significantly degrading quantification accuracy (Bie *et al* 2019, Hufnagel *et al* 2022, Singh *et al* 2025b). However, training a motion-correction network typically requires a large number of subjects. To address this, we employed a self-supervised motion correction technique originally proposed for DWI images, which leverages the STN network with a NCC loss (Lee *et al* 2023). Furthermore, the proposed quantification network was trained with various levels of Gaussian noise (40–46 dB) to account for the complex and variable *in vivo* environments. However, severe noise inevitably compromised quantification accuracy. Therefore, in our study, the robustness was further enhanced by applying the MD-S2S denoising method (Kang *et al* 2024) to *in vivo* ST-MRF images. Since the MD-S2S is based on a self-supervised learning that does not require clean ground-truth, noise could be effectively suppressed without additional scans.

The RF saturation-encoded pulse sequence is vulnerable to field inhomogeneities, which perturb MRF signal profiles and result in significant errors in tissue parameter quantification. Typically, semisolid MTC components have broad spectra due to their very short *T*_2_ relaxation time, making them relatively insensitive to *B*_0_ inhomogeneities. In contrast, APT signals are prone to *B*_0_ inhomogeneities, as amide protons have relatively narrow spectral width and resonate close to the water frequency. In this study, during the ST-MRF data acquisition, several direct water saturation signals were acquired to estimate and correct *B*_0_ field inhomogeneity. Accurate Δ*B*_0_ maps were successfully estimated, showing a high degree of agreement with the WASSR Δ*B*_0_ maps, thereby improving tissue parameter quantification (see supplementary figure S7). The *B*_0_ mapping within the ST-MRF framework eliminates the need for additional scans for *B*_0_ mapping and the subsequent co-registration with MRF images. Accurate correction of *B*_1_ inhomogeneities is essential to quantify CEST parameters. However, in deep learning-based MRF reconstruction, neural networks are constrained to a single MRF schedule corresponding to the training dataset. When B_1_ inhomogeneity is present, the RF saturation field strength (*B*_1_) parameters are scaled for each voxel according to the *rB*_1_ value, and thus, do not ensure consistency of the MRF schedule between training and testing. The proposed dTPQ network was designed for dynamic scan-wise calibration of the *B*_1_ saturation strength, leading to significant improvements in tissue parameter quantification accuracy (also see supplementary figure S7). Thus, the proposed ST-MRF framework, which corrects for both *B*_0_ and *B*_1_ inhomogeneities, could enhance whole-brain ST-MRF imaging, including regions such as the frontal and temporal lobes.

Importantly, assessing quantification resolution (or discrimination power) is crucial to distinguish between pathological and healthy tissues. Our numerical phantom studies demonstrated that the ST-MRF method could differentiate 10 Hz and 10 mm differences in proton exchange rate and concentration of amide protons, respectively. This performance offers sufficient discrimination ability to map amide proton concentration and exchange rates in brain tumor imaging. Accordingly, in the brain tumor study, reduced amide and macromolecule volume fractions were observed in *T*_2_w and FLAIR hyperintense areas, as well as in regions without Gd enhancement, which aligns with findings from a previous CEST-MRF study involving tumor patients (Cohen *et al* 2023). These observations are likely attributed to an increased water volume, particularly in edematous brain tissue, which dilutes the mobile protein fraction relative to the water volume. A study involving a larger patient cohort is needed to more comprehensively elucidate the relationship between MRI-derived tissue parameters and tumor characteristics. Ultimately, the optimal ST-MRF approach could provide an insight into the underlying mechanisms of diseases at a molecular level and offer the potential to detect molecular abnormalities before morphological changes become apparent.

## 6. Conclusion

We developed a deep Bloch equation simulator-based acquisition schedule optimization framework for non-differentiable forward signal models. A deep surrogate model for the Bloch equation solver not only enables rapid simulations but also circumvents the non-differentiability of the forward signal model during gradient calculation in backpropagation. The proposed method provides an optimal acquisition schedule for ST-MRF, based on a three-pool exchange model incorporating the extrapolated super-Lorentzian RF absorption rate of the semisolid macromolecule pool. This approach significantly improves the accuracy of tissue parameter quantification. In addition, *B*_0_ and *B*_1_ inhomogeneities are corrected to accurately estimate the tissue parameter maps. Therefore, the proposed quantitative ST-MRF approach could provide a comprehensive understanding of multiple tissue parameter maps for many pathologies within a clinically acceptable time. This framework can be extended to optimize acquisition schedule for a wide range of MRI sequence protocols, based on various non-differentiable Bloch models.

## Data Availability

The data that support the findings of this study are available upon reasonable request from the authors. Supplementary Material available at http://doi.org/10.1088/1361-6560/ae4285/data1.

## Appendix Theory

**A transient-state three-pool exchange model**

A three-pool exchange model (w: free bulk water proton pool, s: solute proton pool, and m: semisolid macromolecular proton pool) can be used to simulate ST effects, including MTC and CEST, in the presence of proton exchange, relaxation, and RF irradiation. It is assumed that the proton exchange between pool m and pool s is negligible compared to the exchange with pool w. The magnetization vectors of the three pools (***M***^w^, ***M***^s^, and ***M***^m^) can be described using the Bloch–McConnell equations as follows (Zhou *et al* 2004, Woessner *et al* 2005, Sun 2010, Heo *et al* 2016):

\begin{equation*}{\mathrm{d}}{\mathbf{M}}\left(t\right)/{\mathrm{d}}t = {\mathbf{AM}}\left(t\right) + {\mathbf{B}}\end{equation*}

where

\begin{equation*}{\mathbf{M}}(t) = \left[ {\begin{array}{*{20}{c}} {{\mathbf{M}^{\mathrm{w}}}{\mathrm{(}}t)} \\ {{\mathbf{M}^{\mathrm{s}}}{\mathrm{(}}t{\mathrm{)}}} \\ {{\mathbf{M}^{\mathrm{m}}}{\mathrm{(}}t{\mathrm{)}}} \end{array}} \right]\end{equation*}

\begin{equation*}{M^i}\left(t\right) = {\left[ {M_x^i{\text{(t) }}M_y^i{\text{(t) }}M_z^i{\mathrm{(t)}}} \right]^{\mathrm{T}}}\end{equation*}

\begin{equation*}A = \left[ {\begin{array}{*{20}{c}} {{D^{\mathrm{w}}}}&amp;{{E^{{\mathrm{ws}}}}}&amp;{{E^{{\mathrm{wm}}}}} \\ {{E^{{\mathrm{sw}}}}}&amp;{{D^{\mathrm{s}}}}&amp;{{E^{{\mathrm{sm}}}}} \\ {{E^{{\mathrm{mw}}}}}&amp;{{E^{{\mathrm{ms}}}}}&amp;{{D^{\mathrm{m}}}} \end{array}} \right]\end{equation*}

\begin{equation*}{D^i} = \left[ {\begin{array}{*{20}{c}} { - \left({R_{2i}} + {k_{i{\mathrm{w}}}}\right)}&amp;{ - \left(\omega - {\omega _i}\right)}&amp;0 \\ {\left(\omega - {\omega _i}\right)}&amp;{ - \left({R_{2i}} + {k_{i{\mathrm{w}}}}\right)}&amp;{ - \gamma {B_1}} \\ 0&amp;{\gamma {B_1}}&amp;{ - \left({R_{1i}} + {k_{i{\mathrm{w}}}}\right)} \end{array}} \right]\end{equation*}

\begin{equation*}{E^{ij}} = \left[ {\begin{array}{*{20}{c}} {{k_{ij}}}&amp;0&amp;0 \\ 0&amp;{{k_{ij}}}&amp;0 \\ 0&amp;0&amp;{{k_{ij}}} \end{array}} \right]\end{equation*}

\begin{equation*}B = \left[ {\begin{array}{*{20}{c}} {{ }{B^{\mathrm{w}}}} \\ {{ }{B^{\mathrm{s}}}} \\ {{ }{B^{\mathrm{m}}}} \end{array}} \right]\end{equation*}

\begin{equation*}{\mathbf{B}^i} = {\left[ {\begin{array}{*{20}{c}} 0&amp;0&amp;{R_1^iM_0^i} \end{array}} \right]^{\mathrm{T}}}\end{equation*}

where (*M_x_^i^, M_y_^i^*) and *M_z_^i^* are the transverse and longitudinal magnetizations of pool *i*, respectively; *k_ij_* is the proton exchange rate from pool *i* to pool *j; k*_ww_ is given by *k*_ws_ + *k*_wm_; *ω* is the frequency offset of the RF saturation; *ω_i_* ( = *γB*_0_Ω*_i_*, where *B*_0_ is the static magnetic field and Ω*_i_* is the frequency offset in ppm) is the resonance frequency of pool *i* in rad s^−1^, relative to the water resonance; *B*_1_ is the RF saturation strength; *γ* is the gyromagnetic ratio (for *ω*_1_/*B*_1_= *γ =* 267.5 rad μT · s^−1^); *R*_1_*^i^* and *R*_2_*^i^* are the longitudinal and transverse relaxation rates of pool *i*, respectively; and *M*_0_*^i^* is the equilibrium longitudinal magnetization of pool *i.*

Differentiability is lost in the subsequent modeling step. The transverse magnetization of pool *m* is assumed to rapidly reach a steady state (d*M_y_*^m^*/*d*t =* 0) due to its short *T*_2_, which is on the order of microseconds. Therefore, the transverse magnetization of pool m can be rewritten as follows:

\begin{equation*}M_y^{\mathrm{m}} = - \frac{{{\omega _1}T_2^{\mathrm{m}}M_z^{\mathrm{m}}}}{{1 + {{\left[ {T_2^{\mathrm{m}}\left( {{\omega _{\mathrm{m}}} - \omega } \right)} \right]}^2}}} = - \frac{{{R_{{\mathrm{rfm}}}}}}{{{\omega _1}}}M_z^{\mathrm{m}}{\text{ where }}{R_{{\mathrm{rfm}}}} = \omega _1^2\pi {g_{\mathrm{m}}}\left( {\Delta \omega } \right)\end{equation*}

\begin{equation*}\Delta \omega = {\omega _{{\mathrm{off}}}} - {\Delta _{{\mathrm{mw}}}}\end{equation*}

\begin{equation*}{g_{\mathrm{m}}}\left( {\Delta \omega } \right) = \mathop \int \limits_0^{\pi /2} {\mathrm{d}}\theta \sin \theta \sqrt {\frac{2}{\pi }} \frac{{T_2^{\mathrm{m}}}}{{\left( {3{{\cos }^2}\theta - 1} \right)}}{{\mathrm{e}}^{ - 2{{\left( {\frac{{\Delta \omega T_2^{\mathrm{m}}}}{{3{{\cos }^2}\theta - 1}}} \right)}^2}}}\end{equation*}

where RF_rfm_ is the RF absorption rate, which characterizes the decay of longitudinal magnetization due to off-resonance RF saturation; Δ_mw_ denotes the upfield frequency shift of the semisolid macromolecular pool relative to the free bulk water proton pool. In biological tissues, the RF absorption rate is better approximated by a super-Lorentzian lineshape rather than the Lorentzian form (equation (A11)). To mitigate the on-resonance singularity of the super-Lorentzian lineshape, the function is extrapolated from 1 kHz toward its asymptotic limit, in accordance with previous studies (Gloor *et al* 2008, Heo *et al* 2024). Thus, the assumption of a super-Lorentzian lineshape renders the Bloch equation simulator not only computationally expensive but also non-differentiable. The formal solution of the Bloch–McConnell equations (equation (A1)) is described as follows:

\begin{equation*}{\mathbf{M}} = \left({\mathbf{M}_0} + {\mathbf{A}^{ - 1}}{\mathbf{B}}\right){{\mathrm{e}}^{\mathbf{A}{T_{\mathrm{s}}}}} - {\mathbf{A}^{ - 1}}{\mathbf{B}}\end{equation*}

where *T*_s_ is the RF saturation time. In addition, the initial magnetization (***M*_0_**) is altered with the relaxation delay time (*T*_d_) in the absence of RF irradiation as follows:

\begin{equation*}M = \left({M_0}\left(1 - {{\mathrm{e}}^{ - R_1^{\mathrm{w}}{T_{\mathrm{d}}}}}\right) + {A^{ - 1}}B\right){{\mathrm{e}}^{A{T_{\mathrm{s}}}}} - {A^{ - 1}}B.\end{equation*}

Therefore, the longitudinal magnetizations for each pool are simulated based on the given tissue parameters and RF scan parameters, which include saturation strength (*B*_1_), frequency offset (Ω), saturation time (*T*_s_), and relaxation delay time (*T*_d_).
